# IL-10 Rescues CLL Survival through Repolarization of Inflammatory Nurse-like Cells

**DOI:** 10.3390/cancers14010016

**Published:** 2021-12-21

**Authors:** Marcin Domagala, Loïc Ysebaert, Laetitia Ligat, Frederic Lopez, Jean-Jacques Fournié, Camille Laurent, Mary Poupot

**Affiliations:** 1 Centre de Recherches en Cancérologie de Toulouse, Inserm UMR1037, 31037 Toulouse, France; marcin.domagala@inserm.fr (M.D.); ysebaert.loic@iuct-oncopole.fr (L.Y.); laetitia.ligat@inserm.fr (L.L.); frederic.lopez@inserm.fr (F.L.); jean-jacques.fournie@inserm.fr (J.-J.F.); laurent.c@chu-toulouse.fr (C.L.); 2 Université Toulouse III Paul-Sabatier, 31400 Toulouse, France; 3 ERL 5294 CNRS, 31037 Toulouse, France; 4 IUCT-O, 31000 Toulouse, France

**Keywords:** Nurse-like cells, polarization, survival, CLL, IL-10, TNF

## Abstract

**Simple Summary:**

In in vitro co-cultures of CLL cells and nurse-like cells (NLC), protection against apoptosis is only provided by M2-like NLC, and not M1-like NLC. In this study, we propose that fine-tuning of NLC polarization (and therefore survival of leukemic cells) is dictated by a balance between IL-10 and TNF.

**Abstract:**

Tumor-associated macrophages (TAMs) in chronic lymphocytic leukemia (CLL) are also called nurse-like cells (NLC), and confer survival signals through the release of soluble factors and cellular contacts. While in most patient samples the presence of NLC in co-cultures guarantees high viability of leukemic cells in vitro, in some cases this protective effect is absent. These macrophages are characterized by an “M1-like phenotype”. We show here that their reprogramming towards an M2-like phenotype (tumor-supportive) with IL-10 leads to an increase in leukemic cell survival. Inflammatory cytokines, such as TNF, are also able to depolarize M2-type protective NLC (decreasing CLL cell viability), an effect which is countered by IL-10 or blocking antibodies. Interestingly, both IL-10 and TNF are implied in the pathophysiology of CLL and their elevated level is associated with bad prognosis. We propose that the molecular balance between these two cytokines in CLL niches plays an important role in the maintenance of the protective phenotype of NLCs, and therefore in the survival of CLL cells.

## 1. Introduction

Nurse-like cells (NLC) are recognized as tumor-associated macrophages (TAMs) found in the lymphoid organs of chronic lymphocytic leukemia (CLL) patients [1]. This disease is the most common B-cell malignancy in the Western world and is characterized by an accumulation of monoclonal CD5^+^ and mature-appearing B cells in lymphoid tissues and peripheral blood [2]. Numerous studies have shown that CLL cells are especially dependent on their specific tumor microenvironment (TME) and, when cultured in vitro, prone to spontaneous apoptosis. TME of CLL forms a complex medium made up of the extracellular matrix, chemokines, cytokines, and cells including NLC; and has been widely shown to be critical for cancer cell survival, chemoresistance, homing, and proliferation [3,4,5,6,7]. NLC expressing CD68, CD163, and CD206 are found in all tumoral niches [8,9,10]. They form a survival support for CLL cells, mediated by cell contacts involving different surface molecules such as LFA-3 or galectin-1 [7,11,12] and, controversially, CD31 [13,14,15]. Moreover, NLC produce pro-survival soluble factors, such as: BAFF, APRIL, SDF-1 (CXCL12), or BDNF [16,17], and can also stimulate the release of CCL3 and CCL4 by leukemic cells to further strengthen TME [18]. It has been shown that soluble molecules, such as HMGB1 or CSF-1, were involved in NLC differentiation from blood monocytes, [8,19], but that cell contacts between monocytes and CLL cells are also essential [1,10]. However, TAMs, like other macrophages, are highly plastic and readily respond to signals from the microenvironment by fine-tuning their polarization status. Thus, their phenotype will depend on the balance between various pro-M1 factors, such as TNF, IFN-γ, or pro-M2 factors including IL-4, IL-13, or IL-10 [20]. NLC were clearly defined to be closer to the M2 end of the spectrum regarding their phenotype and their pro-survival properties [21] (for review). Unexpectedly, both TNF and IL-10 are implicated in the pathophysiology of CLL, levels of which have been correlated with an adverse prognosis in vivo [22,23,24]. Indeed, TNF has been listed as an important factor for leukemic proliferation and survival [22,25]. This is not in agreement with the fact that TNF favours the M1 macrophage phenotype, despite the current view of NLC being M2-polarized in the TME. On the other hand, IL-10 thwarts anti-tumoral immune responses, inhibits pro-inflammatory cytokines like TNF, and increases the level of pro-survival molecules such as ICAM-1 on the surface of CLL cells [26,27,28].

So far, no study has investigated the simultaneous influence of TNF and IL-10 on NLC-mediated protection of CLL cells. We propose here that IL-10 counterbalances the pro-M1 effect of TNF on NLC, thus preserving their pro-niche capacity. We believe that these experiments are useful to understand the impact of TNF and IL-10 on pro-tumoral phenotypes of NLC.

## 2. Materials and Methods

### 2.1. Patient’s Samples

Peripheral blood samples from CLL Patients were obtained from the Hematology Department with informed consent and referenced in the INSERM cell bank. According to French law, the INSERM cell bank has been registered with the Ministry of Higher Education and Research (DC-2013-1903) after being approved by an ethics committee (Comité de Protection des Personnes Sud-Ouest et Outremer II). Clinical and Biological annotations of the samples have been reported to the Comité National Informatique et Liberté (the Data Processing and Liberties National Comittee). Blood samples were collected from previously untreated patients. PBMC from CLL patients’ blood were isolated by density-gradient centrifugation on Ficoll-Paque^TM^ PLUS (GE Healthcare, Sweden). For all experiments except co-culture, fresh PBMC were used. The remaining cells were frozen in Cryostor CM10 (STEMCELL Technologies, France) and stored in liquid nitrogen.

### 2.2. CLL Cells Isolation

CLL cells were isolated from fresh or frozen PBMC using a negative selection kit—EasySep™ Human B Cell Enrichment Kit II Without CD43 Depletion (STEMcell, Saint Égrève, France), according to the manufacturer’s instructions. Purity and viability of the cells after isolation were measured by flow cytometry following staining with CD5/CD19 antibodies and Annexin-V/7-AAD, respectively. For each experiment both values exceeded 95%.

### 2.3. Cell Cultures

The whole PBMC and isolated CLL cells cultures were cultured in a complete medium: RPMI 1640 with GlutaMAX (Gibco, France) supplemented with 10% of FBS (Life Technologies, France) and 100 µg/mL of penicillin/streptomycin (Sigma-Aldrich, France). Cells were plated at high density (10 × 10^6^ cell/mL) in tissue culture treated plates (Corning, USA), and maintained at 37 °C in a humidified atmosphere with 5% CO_2_. In experiments to evaluate the NLC phenotype, cells were cultured in a 4 mL/well of 6-well plates, and to evaluate CLL cells viability, cells were cultured in a 200 µL/well of a 96-well plate or in a 400 µL/well of a 48-well plate in the case of fluorescence microscopy imaging.

### 2.4. Cytokine Treatment

In order to evaluate the effect of cytokines on the NLC phenotype and CLL cells survival, cells were treated at the indicated time points with 10 ng/mL of human TNF or 50 ng/mL of IL-10 (Miltenyi Biotec, France).

#### 2.4.1. Blocking Antibodies

Blocking antibodies against human TNF or IL-10, and relevant isotype controls were purchased from BD Pharmingen (France). To evaluate the effect of TNF or IL-10 depletion on CLL cells viability and NLC phenotype, PBMC were treated at day zero with a single dose of antibodies alone or in combination at 10 µg/mL, with or without the addition of 10 ng/mL of TNF. The effect of blocking antibodies on CLL cells viability or NLC phenotype was measured on day 12 by flow cytometry.

#### 2.4.2. Co-Culture Experiments

Co-culture experiments were used to imitate CLL cells contact with resident NLC after re-entering the lymph node.

NLC were generated as described above. After 12 to 14 days of culture non-adherent PBMC were separated from adherent NLC by gentle pipetting and washed twice with PBS at room temperature. Subsequently, NLC were co-cultured with purified CLL cells at 10 × 10^6^ cells/mL in 200 µL of complete medium, supplemented with TNF or IL-10. To access basal survival of cancer cells, CLL cells alone were cultured in parallel. CLL cells were co-cultured with NLC from the same (autologous) or different (heterologous) patient. After five days of co-culture, CLL cells viability was measured by flow cytometry using Annexin-V/7-AAD staining.

### 2.5. Flow Cytometry Analysis

Antibodies against CD5-PC7, CD19-BV421, CD14-PC7, CD16-PE, CD64-PE, CD71-APC-Cy7, CD86-BV421, CD163-FITC, CD169-APC, CD206-BV421, CD209-FITC, and relevant isotype controls were purchased from Sony Biotechnology Europe. 7-AAD and Annexin-V-FITC viability kit were purchased from Miltenyi Biotec, France. Data were acquired on an LSRII flow cytometer (BD Biosciences, France). The obtained results were further analyzed by Flow Logic 700.2A (Inivai Technologies, Australia).

#### 2.5.1. CLL Cells Analysis

On day seven or 12 of culture, floating cells were separated by gentle pipetting and incubated with Human BD Fc Block™ (2.5 µg/mL) in flow cytometry buffer (2% FBS in PBS) for 15 min at 4 °C, followed by staining with 1 µg/mL of CD5 and CD19 antibodies for 20 min at 4 °C. After washing, cells were stained with Annexin-V-FITC according to manufacturer protocol. Following the Annexin-V staining, cells were re-suspended in Annexin-V buffer, containing 7-AAD (0.8 µg/mL) and immediately analyzed by flow cytometry.

#### 2.5.2. NLC Analysis

After 12 or 14 days of CLL PBMC culture, floating cells were harvested and NLC were washed twice with PBS and incubated with 1 mL of Accutase (Biolegend, France) for 20 min in 37 °C. Subsequently, 0.5 mL of FBS was added and cells were further detached with gentle scrapping (Sarstedt, USA). Both floating and adherent cells were further combined, washed in PBS, re-suspended in flow cytometry buffer containing 2.5 µg/mL of Human BD Fc Block™ (BD Biosciences, France) and incubated for 15 min at 4 °C. Subsequently, cells were stained with antibodies at saturating concentrations, for 20 min at 4 °C. After washing, cells were re-suspended in PBS, containing 7-AAD (0.8 µg/mL) and immediately analyzed by flow cytometry.

### 2.6. Fluorescence Microscopy Imaging

PBMC for fluorescence microscopy analysis were plated in 48-well plates in 400 µL of the complete medium at 10 × 10^6^ cells/mL to allow maturation of NLC. Cytokine treatments and removal of the floating PBMC were cultured according to the co-culture paragraph. Additionally, NLC and isolated CLL cells were stained with specific fluorescent dyes for subsequent visualization of contact between CLL cells and NLC or measurements of phagocytosis during the culture.

#### 2.6.1. Stainings

*Visualization of contact between CLL cells and NLC.* After removal of floating cells, NLC were stained with mitochondrial MitoView633 (Biotium, USA) or Cell MASK Deep Red plasma membrane dye (Thermo Fisher Scientific, France). Purified CLL cells were stained with cytoplasmic CellTracker Orange CMTMR (Sigma-Aldrich, USA) or CellTrace™ CFSE Cell Proliferation Kit (Thermo Fisher Scientific, France) dye according to the manufacturer’s instructions.

*Phagocytosis assay.* During in vitro CLL culture, NLC can phagocyte dying CLL cells in a process called efferocytosis. To evaluate the phagocytosis capabilities of NLC after cytokine treatment, purified CLL cells were stained with pHrodo dye (Thermo Fisher Scientific, USA). The fluorescence of pHrodo is dependent on pH and increases significantly with acidification of environment (such as in the case of a phagocytosis event). For the pHrodo staining, each 5 × 10^6^ of purified CLL cells were diluted in 500 µL of PBS and mixed with 500 µL of staining buffer (0.1 M of sodium bicarbonate, pH 8.5) containing freshly diluted pHrodo to the final concentration of 20 µg/mL. Cells were incubated in a multiwells plate for 2 h at 37 °C and 5% CO_2_. Subsequently, cells were collected and washed twice with a cold complete medium. Next, cells aggregates were removed by passing through a 40 µm nylon filter (Miltenyi Biotec, France), counted with a haemocytometer (Marienfeld, Germany), and plated at 2 × 10^5^ cells/mL of complete medium containing 1 nM of Hoechst 33342 (Life Technologies, USA) with pre-stained NLC.

#### 2.6.2. Image Acquisition

Following the staining, the plates were mounted in Operetta CLS High-Content Analysis System (PerkinElmer, France) equipped with an automated spinning disk fluorescence confocal microscope and analyzed with a 20× objective. Cells were maintained at 37 °C and at 5% of CO_2_. Cells morphology and distribution were visualized using bright field (BF) imaging. Depending on the staining: violet, green, orange or red channels were used to visualize cell nuclei, mitochondria, cytoplasm or plasma membrane of the cells. For each well, multiple fields were analysed and images were acquired every 15 min for at least 3 h.

#### 2.6.3. Image Analysis

Calculation of changes in pHrodo intensities within NLC were evaluated by Columbus software (PerkinElmer, France). Briefly, NLC were segmented based on the size, nuclei detection, and plasma membrane staining. The pHrodo intensity values within each NLC cell region were extracted and changes in the pHrodo signal intensities were calculated by subtracting fluorescence values at the first time point from the fluorescence values at the last time point (3 h).

### 2.7. Statistical Analysis

**Figure 1:**
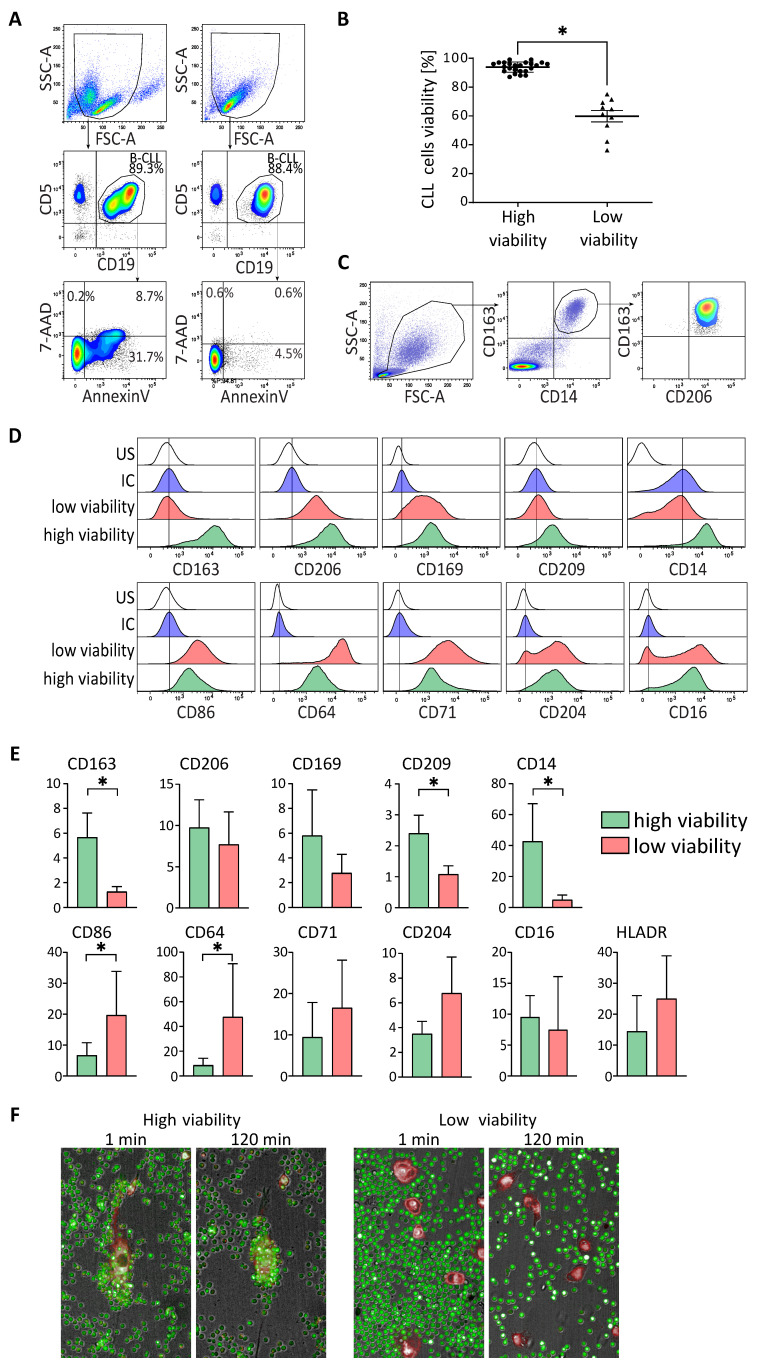
Non protective NLC for CLL cells harbour an M1-like phenotype. (**A**,**B**). Flow cytometry analysis of the percentage of viability (Annexin V/7-AAD negative cells) of CLL cells from cultures of PBMC from CLL patients after 14 days. (One representative experiment (**A**) and data for PBMC from 35 CLL patients separated in two groups, one with high CLL cells viability and one with low CLL cells viability (**B**)). (**C**–**E**) Surface markers expressed by NLC analysed by flow cytometry at 14 days of cultures of PBMC from CLL patients with low (red) or high (green) in vitro CLL cells viability, comparing to the unstained (US: white) and the isotypic (IC: blue) controls (**D**) one representative experiment; (**E**) compilation of MFI ratios (marker/isotypic control) of five donors. (**F**) Fluorescence imaging of co-cultures of NLC (red staining) and CLL cells (green staining), at 1 min and 120 min, for patients with high (left) and low (right) in vitro viability of CLL cells. * indicates *p* < 0.05.

**Figure 2:**
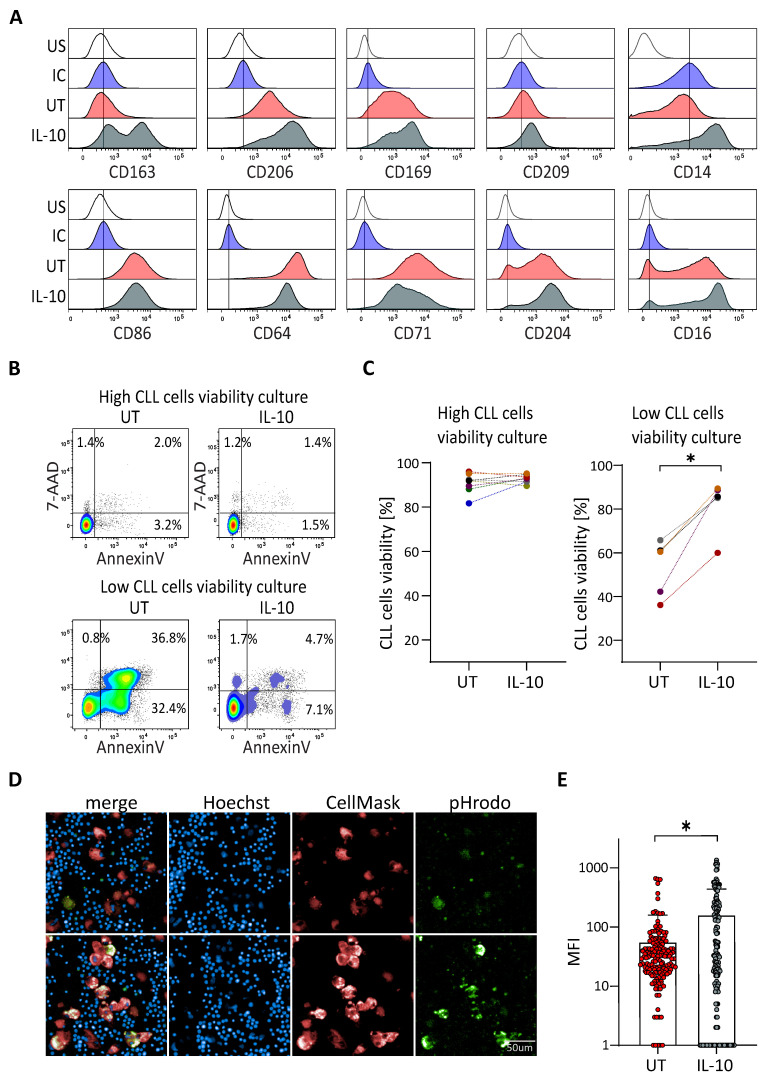
IL-10 rescues viability of CLL cells from patients with low protective NLC. (**A**) Surface markers expressed by NLC analysed by flow cytometry at 14 days of cultures of PBMC from CLL patient with low in vitro CLL cells viability, untreated (UT: red) or treated with IL-10 (grey), comparing to the unstained (US: white) and the isotypic (IC: blue) controls. Representative histograms from five independent experiments. (**B**,**C**) Percentage of the CLL cells viability at 14 days of culture of PBMC from CLL patient with low or high in vitro CLL cells viability untreated (UT) or treated with IL-10 (**B**) one representative experiment; (**C**) eight and five independent experiments for high viability and low viability cultures respectively). (**D**,**E**) Phagocytosis of CLL cells (blue dots) by NLC (red) visualized (**D**) upper: untreated; lower: IL-10 treated and quantified (**E**) thanks to the green pHrodo fluorescence. * indicates *p* < 0.05.

**Figure 3:**
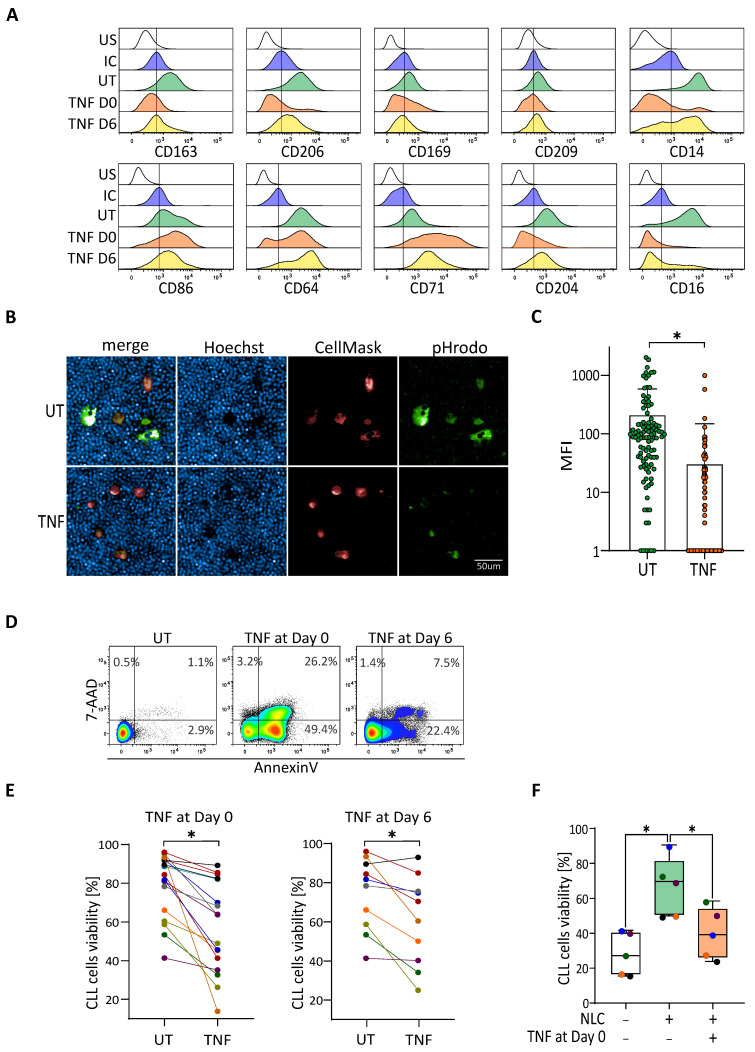
TNF depolarizes protective NLC, leading to a decrease of CLL cells viability. (**A**) Surface markers expressed by NLC analysed by flow cytometry at 14 days of CLL’s PBMC cultures incubated or not (UT: green) with TNF at day zero (orange) or at day six (yellow), compared to the unstained (US: white) and the isotypic (IC: blue) controls. Representative histograms from eight independent experiments. (**B**,**C**) Phagocytosis of CLL cells (blue dots) by NLC (red) is visualized. (**B**) upper: untreated; lower: TNF treated and quantified (**C**) thanks to the green pHrodo fluorescence. (**D**,**E**) Percentage of CLL cells viability at 14 days of culture of CLL PBMC-incubated or not (UT) with TNF at day zero or at day six. (**D**) one representative experiment. (**E**) A total of 19 independent experiments for TNF treatment at day zero and 10 independent experiments at day six. (**F**) Co-culture experiments: percentage of the CLL cells viability cultivated alone (white) or co-cultivated with autologous control NLC (untreated, green) or depolarized NLC (previously treated with TNF; orange). * indicates *p* < 0.05.

**Figure 4:**
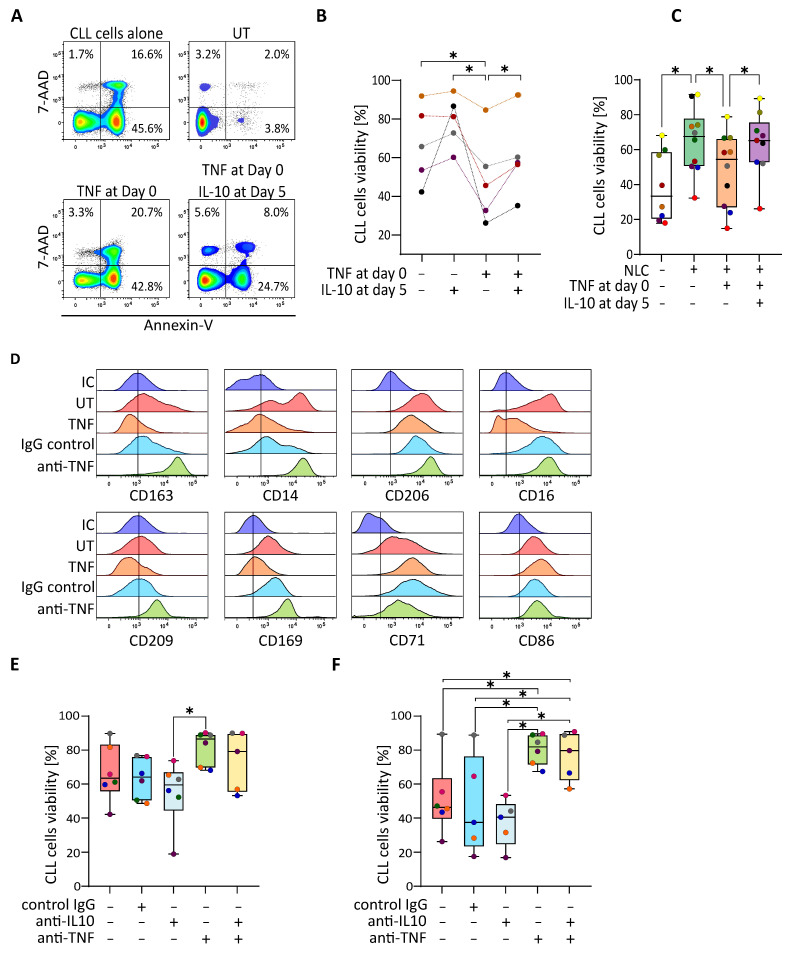
IL-10 repolarizes TNF-depolarized NLC which induce the increase of CLL cells viability. (**A**,**B**) Flow cytometry analysis of the viability (Annexin V/7-AAD negative cells) of CLL cells from cultures of CLL’s PBMC incubated or not (UT) with TNF at day zero following by treatment or not with IL-10 at day five. (**A**) one representative experiment; (**B**) five independent experiments. (**C**) Percentage of the CLL cells viability alone (white) or in a CLL’s PBMC culture without treatment (green) or in co-culture with autologous depolarized NLC previously treated with TNF (orange) or with autologous repolarized NLC (treated with TNF at day zero then with IL-10 at day five; purple) (14 independent experiments). (**D**) Surface markers expressed by NLC analysed by flow cytometry at 14 days of CLL’s PBMC cultures incubated or not (low viability, UT: red) with TNF at day zero (orange) or with TNF plus an IgG control (blue), or with TNF plus an anti-TNF antibody (green), compared to the isotypic control (IC: dark blue). (**E**,**F**) Percentage of the CLL cells viability at day 14 in CLL’s PBMC cultures untreated (**E**) or treated with TNF at day zero (**F**) without antibodies (red) or in the presence of IgG negative control (blue) or anti-IL-10 antibody (grey) or anti-TNF antibody (light green) or the combo anti-IL-10 antibody plus anti-TNF antibody (pale yellow) (nine independent experiments). * indicates *p* < 0.05.

The Mann–Whitney U test was used for comparison of viability of CLL cells from different cultures (Figure 1B) or MFI ratios of surface markers on NLC (Figure 1E). Paired t-test (Figure 2C,E and Figure 3C) or Wilcoxon test (Figure 3E) were used to determine the differences in viability of CLL cells or MFI ratio in phagocytosis assays. One-way ANOVA with Geisser–Greenhouse correction (Figure 3F and Figure 4B,C,E,F) was used to compare the results after various treatments.

Prior to statistical analyses, the flow cytometry data (presented on Figure 1E), was first transformed into an MFI ratio. Results of the bar plots are shown as mean ± SD. The lines in the middle of the boxes for box and whiskers plots represent median values. All statistical analyses were performed using Graph Pad Prism 9.1.2. The p-values below 0.05 were considered statistically significant.

## 3. Results

### 3.1. CLL Cells Viability Depends on M2-like NLCs In Vitro

The culture of CLL cells in the presence of NLC typically allows the cancer cells to maintain high viability in vitro. To our surprise, in some of the CLL PBMC cultures, we observed poor survival of CLL cells, despite the presence of NLC. (Figure 1A,B). In order to elucidate this phenomenon, we decided to analyse the phenotype of NLC by flow cytometry, especially as we didn’t observe clear connections between mutational status and lymphocytosis of the patients, and the viability of CLL cells in vitro. To do that, we cultured CLL PBMC for 14 days to allow for the outgrowth of NLC, thanks to contact between monocytes and cancer cells. Then, we investigated the phenotype of NLC by flow cytometry. Classically, NLC are characterized by M2-like phenotype with a high expression of CD163 and CD206 (Figure 1C and Supplementary Figure S1). We further enriched the analysis with several myeloid markers to better distinguish potential differences between NLC from PBMC with low CLL cells in vitro viability (noted: low viability) and from PBMC with high CLL cells in vitro viability (noted: high viability), In comparison to isotypic controls, expression of CD163, CD206, CD169, CD209, and CD14 was higher for NLC from samples with high viability (Figure 1D: green) compared to expression by NLC from samples with low viability (Figure 1D: red). Moreover, expression of CD86, CD64, CD71, and in a smaller proportion of CD204, was higher for NLC from samples with low viability (Figure 1D: red) compared to expression by NLC from samples with high viability (Figure 1D: green). This was observed with at least six different donors from each group (Figure 1E). To confirm that the NLC phenotype analyzed from these two kinds of samples translates into their functionality, we tested the propensity of NLC to attract CLL cells in a protective manner. After 14 days of high or low viability PBMC cultures, floating cells were removed and the remaining NLC were stained with a plasma membrane fluorescent dye. Subsequently, CLL cells were isolated from the frozen PBMC sample, stained with cytoplasmic fluorescent dyes and mixed with NLC. Cells were then visualized by live video-microscopy using an Operetta system. The acquired pictures clearly show a strong grouping of CLL cells around NLC from samples with high viability already after one minute which progresses further until 120 min into the experiment, while NLC from samples with low viability did not induce this phenomenon (Figure 1F and Supplementary Figure S2 for video).

These results prove that NLC from high viability CLL cultures not only display an M2-like phenotype but are also able to attract and facilitate contact with cancer cells, which has been previously shown as the hallmark of their protective functions. On the contrary, NLC from low viability CLL cells cultures are characterized by an M1-like phenotype, with no capacity to attract CLL cells.

### 3.2. IL-10 Rescues the Viability of CLL Cells in Patient Samples without Protective NLC

IL-10 is known to be an immunosuppressive agent, favouring the M2 phenotype of macrophages. We wanted to know whether this cytokine, naturally produced by CLL cells, could be involved in the protective effect of NLC for CLL cells. PBMC from patients with low CLL cells in vitro viability were cultivated with or without IL-10 for 14 days. Subsequently, the phenotype of NLC was analysed by flow cytometry. Treatment with IL-10 induced an increase of CD163, CD206, CD169, CD14 and weakly of CD209, and a decrease of CD64 and CD71 (Figure 2A). This was in agreement with a switch of the NLC phenotype towards an M2-like phenotype close to that of NLC generated in culture with high CLL cells viability. To go further, the effect of IL-10 was also evaluated on CLL cells viability. IL-10 was shown as having no effect on CLL cells alone (Supplementary Figure S3) or on the viability of CLL cells from samples known to have a high viability. However, IL-10 significantly increased the CLL cells viability in samples with low viability (Figure 2B,C). We then compared phagocytosis capacities of NLC from low viability cultures and NLC from cultures treated with IL-10. Results showed that NLC from IL-10-treated cultures displayed a significantly higher propensity to phagocyte dying CLL cells (efferocytosis) compared to untreated NLC (Figure 2D,E and Supplementary Figure S4 for video).

Thus, the addition of IL-10 in the culture induces an M2-like phenotype of NLC, which in turn leads to increased survival of CLL cells viability.

### 3.3. TNF-Induced Depolarization Abrogates Pro-Survival Functionality of NLC

TNF is known as a strong pro-inflammatory cytokine polarizing macrophages towards an M1-like state, but has also been linked to an increase of CLL cells viability [22,25]. Thus, we asked the question of whether the treatment of CLL’s PBMC cultures with TNF could induce a modification of the NLC phenotype, leading in turn to a decrease in CLL cells viability. This could potentially mimic the phenotype of low viability CLL cultures characterized previously. PBMC from blood samples of CLL patients were cultivated for 14 days in the presence of TNF added either at day zero or at day six of the culture for each sample. NLC phenotype and CLL cells viability were analysed by flow cytometry, while the phagocytic properties of NLC were evaluated by fluorescent microscopy. As shown in Figure 3A, TNF added at the beginning of the culture induced a strong decrease of the expression of CD163, CD206, CD169, CD14, CD16, and CD204; and an increase of CD86, CD64, and CD71. The addition of TNF at day six induced the analogous, although less pronounced, changes for the previously mentioned surface markers (Figure 3A). The depolarization of NLC with other M1 agents, such as IFN-γ and LPS, also changed the final polarization of NLC, but with a distinct pattern and effect on the viability of CLL cells. On the other hand, treatment with IL-10 led to a slight enforcement of the M2 phenotype of NLC (Supplementary Figure S5). We then compared phagocytosis capacity of NLC from high viability cultures and NLC from cultures treated with TNF. Results showed that NLC from untreated cultures displayed a significantly higher propensity to phagocyte dying CLL cells (efferocytosis) compared to NLC treated with TNF (Figure 3B,C). Analysis of the percentage of the viability of CLL cells was done by staining with 7-AAD and Annexin V (Figure 3D). Treatment of the cultures with TNF at day zero or day six induced a significant decrease of the CLL cells viability compared to cultures without treatment (Figure 3E). No direct toxic effect of TNF was observed on CLL cells viability (Supplementary Figure S3). To be sure that the decrease of CLL cells viability was strongly due to the depolarisation of NLC, we performed co-culture experiments. PBMC from the same patients were cultivated for 12 to 14 days in the presence or absence of TNF. Subsequently, floating cells were removed and NLC were further co-cultured with autologous CLL cells isolated from frozen PBMC vials. The viability of isolated CLL cells was then analysed after five days of co-culture. As expected, the viability of CLL cells cultured alone was significantly decreased compared to the condition with M2-like NLCs. Interestingly, the CLL cells viability from the cultures treated with TNF was comparable to that of CLL cells cultured alone, signifying the lack of protective effect of NLC on leukemic cells (Figure 3F).

TNF is therefore able to depolarize protective M2-like NLC into non-protective M1-like NLC.

### 3.4. IL-10 Repolarizes TNF-Depolarized NLC to Recover the Protective Effect on CLL Cells

We then wondered whether IL-10 could induce the repolarization of TNF-depolarized NLC, which in turn, would result in an increase of CLL cells viability. PBMC from CLL patients were therefore cultivated in the presence of TNF at day zero, then IL-10 was added or not at day five. The CLL cells viability analysis by flow cytometry showed, as expected, a decrease in the viability of cultures treated with TNF, which could be partially countered by co-treatment with IL-10 at day five (Figure 4A,B). At the same time, we showed that low viability CLL cells cultures (Figure 4B: black, purple and green samples) could also be rescued by the addition of IL-10 at day five. Going further, we performed the same experiment of co-culture of freshly isolated CLL cells and TNF-depolarized NLC (TNF at day zero) or IL-10-repolarized NLC (TNF at day zero then IL-10 at day five). As predicted, the presence of protective M2-like NLC in the culture increased the viability of CLL cells, compared to the monocultures of cancer cells. At the same time, the co-culture with TNF-depolarized NLC showed a decrease of cancer cells viability, comparable to the culture of CLL cells alone (Figure 4C: orange block). However, the percentage of CLL cells viability was increased when CLL cells were co-cultivated with IL-10-repolarized NLC (Figure 4C: purple block). To reinforce the message of relevance of IL-10 on the protective function of NLC and thus CLL cells survival, we used blocking antibodies against IL-10 and TNF. First, the phenotype of M1-like NLC cultivated in the presence of anti-TNF or IgG control was analysed by flow cytometry. Expression of CD163, CD14, CD206, CD209, and CD169 which were further decreased by the presence of TNF in the culture, were restored in the presence of anti-TNF compared to the cultures with the IgG control, while expression of CD71 was slightly decreased (Figure 4D). The expression of CD16 and CD86 were similar in the conditions with anti-TNF or IgG control. The percentage of CLL cells viability in the presence of blocking antibodies was also evaluated, revealing that blocking IL-10 in the culture induced a decrease of the viability while blocking TNF increased it slightly (Figure 4E). These effects were even more pronounced if cells were treated with TNF at the beginning of the culture, when the addition of the anti-TNF blocking antibody led to an increase of the viability percentage, while blocking IL-10 led to a small decrease depending on the sample (Figure 4F).

## 4. Discussion

CLL cells have been shown to be resistant to in vitro apoptosis, in the majority of cases, if cultivated in the presence of NLC [29]. However, in some cases CLL cells display a decrease in survival, even in the presence of NLC. We demonstrated here that NLC obtained from PBMC of these patients can be considered as non-protective for CLL cells, displaying an M1-like phenotype, with a low capacity to attract leukemic cells, and are unable to perform efferocytosis, i.e., phagocytosis of dying cells. However, addition of IL-10 to the cultures of PBMC from these patients led to a significant increase in CLL cells viability. The role of IL-10 in mediating leukemic survival is still controversial, implying a possible involvement in CLL cells maintenance or cell death. IL-10 was involved as provoking apoptosis by decreasing Bcl-2 protein levels or activating STAT1 protein [30,31]. On the contrary, IL-10 was given as a pro-survival cytokine for CLL cells acting as an autocrine growth factor and mediating pro-survival signals through the activation of STAT3 [32,33,34]. However, nobody asked the question of a possible indirect effect of IL-10 on CLL cells, notably via the microenvironment such as NLC.

Given that IL-10 is a pro-M2 cytokine for myeloid cells and that protective NLC display an M2-like phenotype, this cytokine could maintain and/or polarize NLC towards an M2 protective state to further support leukemic survival. To answer this question, we first generated a model which could mimic cultures with low CLL cells viability. We showed that the addition of TNF in cultures of PBMC from patients with high in vitro CLL cells viability led to a preclusion of the M2 polarization of blood monocytes into NLC when added at day zero, and to a depolarization of maturating NLC when added at day six of the culture. Even though TNF was shown in some studies as increasing CLL cells viability and correlated with a bad prognosis in patients [22,25], we showed here that it is also able to block or depolarize the M2 protective phenotype of NLC leading to a decrease in leukemic viability (Figure 3). M2 markers such as CD206, CD204, CD169, CD163, and CD16 were abrogated with TNF at day zero and highly decreased with the addition of TNF at day six of the culture with an increase of M1 markers, such as CD86, CD64 and CD71. At the same time, TNF treatment led to a significant decrease of the efferocytosis capacity of NLC. To further exclude the possibility that the effect of TNF might be mediated by other cells present in CLL PBMC culture, we performed experiments with mature NLC co-cultured with freshly isolated CLL cells, in an autologous manner. Co-cultures with TNF- treated NLC led to a decrease in the survival of CLL cells, proving that depolarization of NLC decreases their protective effect. Interestingly, IL-10 was shown here to restore the survival of CLL cells from low viability PBMC cultures by inducing M2 polarization of NLC, and also to rescue CLL cells in PBMC cultures treated with TNF. Furthermore, co-culture experiments with freshly isolated CLL cells and TNF depolarized NLC further repolarized by IL-10 displayed the restoration of protective NLC capacities. Thus, we demonstrated here for the first time that non-protective NLC with an M1-like phenotype can be reoriented towards an M2 protective phenotype thanks to IL-10. This was in agreement with the indirect action of IL-10 on the CLL cells viability via NLC.

Moreover, blocking of TNF in CLL’s PBMC cultures increased CLL cells viability, in particular for samples from patients with low in vitro viability. Interestingly, blocking of IL-10 in CLL’s PBMC cultures could decrease the CLL cells viability for samples previously displaying high survival of CLL cells, and further decrease survival in initially low viability cultures, especially upon additional treatment with TNF.

All these results, combined with multiple research listing TNF as an important factor for CLL progression, suggest that the TNF and IL-10 interplay constitutes an important axis, allowing both activation of CLL cells by TNF, while at the same time, IL-10 is preserving the M2 protective polarization state of NLC. The M2 polarization of NLC shown by high CD163 staining in lymph nodes was indeed correlated with progressive disease [6]. IL-10 and TNF produced by CLL cells and others in the in vitro culture of PBMC from CLL patients may therefore have an impact on the NLC phenotype and CLL cells viability. IL-10 favours the M2-like protective NLC state and counterbalances the M1-agent TNF, preserving the pro-tumoral effect of NLC on leukemic cells.

Even if in vitro cultures of PBMC from CLL patients do not exactly reflect the CLL microenvironment, it can be sufficiently representative to appreciate the interaction between CLL cells and NLC. No work in the literature has reported concentrations of TNF or IL-10 in lymph nodes. However, TNF was shown as produced by T cells, CLL cells and macrophages in CLL patients [25]; and TNF released by CLL cells was proposed as one of the reasons for dysfunctional hematopoiesis in CLL [35]. IL-10 was also shown to be produced by CLL cells. Interestingly expression of this cytokine was shown to be upregulated by CD5, which is present on the surface of leukemic cells [36]. Additionally, CD5 expression is modulated, dependent on the life cycle of CLL cells. Its expression is increased while CLL cells reside in lymph nodes and, after returning to peripheral blood, CD5 level is gradually downregulated. It would further suggest, that CLL cells express the most of IL-10 while in the lymph nodes, where this cytokine could not only support proliferation of the CLL cells, but also preserve the pro-tumoral polarization state of NLC [36]. In addition, high levels of TNF and IL-10 were measured in the serum of high risk groups of CLL patients [23] with a median of 5.04 pg/mL for IL-10 compared to zero for healthy donors [27,37,38] and 17 to 34 pg/mL for TNF, level for healthy donors being at 5 pg/mL [39]. It is impossible to extrapolate this information to the lymph nodes compartment but we could speculate that cells of TME are continuously producing and using these cytokines. Levels of IL-10 and TNF in serum and/or in lymph nodes of patients could be therefore linked to the aggressiveness of the disease, and directly linked to NLC polarization, which has a high impact on leukemic cells viability. The amount of these cytokines and more precisely the proportion of IL-10 / TNF produced in the TME can be responsible for the polarization of NLC on which CLL cells viability depends. Classical therapies can also modulate the proportion of these cytokines. Indeed, treatment of patients with ibrutinib enforces M2 phenotype of NLC with exacerbating M2 markers expression, increasing their immunosuppressive profile [34]. Moreover, in vitro treatment of NLC with ibrutinib induces the protection of CLL cells from drug-induced apoptosis partially through the secretion of IL-10 [34]. This results in further support targeting of IL-10 to potentially decrease pro-tumoral function of NLC and TME. Anti-IL-10 in combination with classical therapies in CLL could be beneficial as shown in other diseases such as leukaemia or melanoma [40,41]. In addition, all conventional drugs used in the treatment of CLL could be applied in cultures of CLL cells in vitro to document the types of molecules / cytokines that could be released that induce the depolarization of M2 protective NLC in M1 non-protective NLC.

These results also enforce the possibility of targeting TNF that is associated with disease progression [22,42]. Blocking of TNF could therefore lead to stabilization of the disease, or at least to partial resolution of inflammation. This could be followed by anti-IL10 treatment, combined with targeting of the CLL microenvironment, particularly NLC, to decrease the survival of remaining cancer cells.

## 5. Conclusions

In this study, we showed that NLC obtained from PBMC of patients with a low in vitro viability of CLL cells can be considered as non-protective for CLL cells, displaying an M1-like phenotype. We also demonstrated that these non-protective NLC or TNF-depolarized NLC can be reoriented towards an M2 protective phenotype thanks to IL-10, confirming the importance of IL-10 in the CLL progression.

## Data Availability

Not applicable.

## Supplementary Materials

The following are available online at https://www.mdpi.com/article/10.3390/cancers14010016/s1, Figure S1: NLC display a phenotype close to M2 macrophages. Figure S2: Video microscopy from a sample with high CLL cells viability. Figure S3: TNF and IL-10 have no effect on the viability of isolated CLL cells. Figure S4: video microscopy with IL-10. Figure S5: TNF, IFN-γ and LPS depolarize NLC toward a M1-like phenotype.
