# Supplementary material for: IL-10 Rescues CLL Survival through Repolarization of Inflammatory Nurse-like Cells
Source: Cancers (Basel). 2021 Dec 21;14(1):16. doi: 10.3390/cancers14010016 (PMC8750769; doi:10.3390/cancers14010016)
Supplement: Supplementary file 1 [file cancers-14-00016-s001.zip › cancers-1451830-supplementary.pdf]

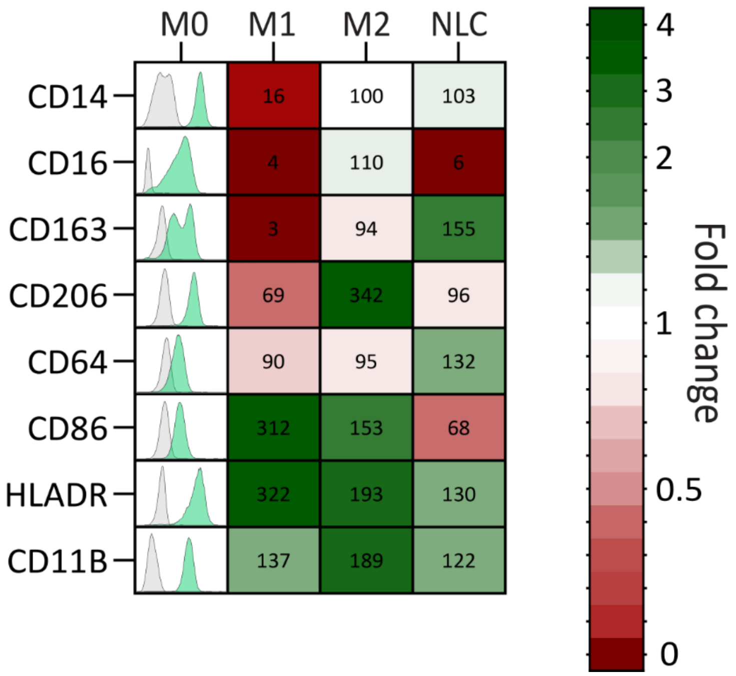

Figure S1: NLC display a phenotype close to M2 macrophages. Monocyte-derived macrophages (MDM) were obtained from $CD14^{+}$ monocytes isolated from normal human PBMC. M0-MDM were obtained by activation of monocytes with CSF-1 for 8 days. M1-MDM were obtained by activation of monocytes with GM-CSF for 6 days, followed by polarization with IFN-γ for 48h and LPS for 30h. M2-MDM were generated by activation of monocytes with CSF-1 for 6 days and subsequent polarization with IL-4 and IL-13 for 48h. NLC were obtained by culture of CLL PBMC at $10x10^{6}$ cells/mL for 14 days, as described previously. After maturation cells were analyzed by flow cytometry. MFI ratio (marker/isotypic control) were normalized as percentage of change of marker signal in relation to basal signal – M0 MDM. Normalization has been performed for each sample separately, followed by calculation of mean of all experiments (4 donors).
(green: expression increased compared to UT; white: baseline expression in UT conditions; red: expression decreased compared to UT).

Video file: grouping for CLL high viability

Figure S2: Video microscopy of co-cultures of NLC (red) and CLL cells (green) from a sample with high CLL cells viability.

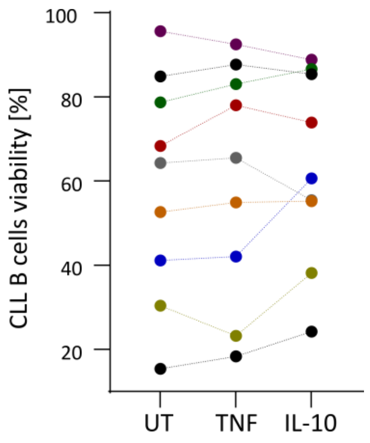

Figure S3: **TNF and IL-10 have no effect on the viability of isolated CLL cells.** CLL cells isolated from PBMC from CLL's patients were cultivated 12 days in the presence of TNF or IL-10. CLL cells viability was analyzed by flow cytometry (7-AAD and Annexin V) and the percentages of viable cells were reported for all conditions: untreated (UT), TNF treated and IL-10 treated (9 independant experiments).

Video files: phagocytosis IL-10

Figure S4: video microscopy of co-cultures of NLC (red) with CLL cells (green) from a sample with low CLL cells viability with IL-10 to show phagocytosis visualized by the green fluorescence in NLC.

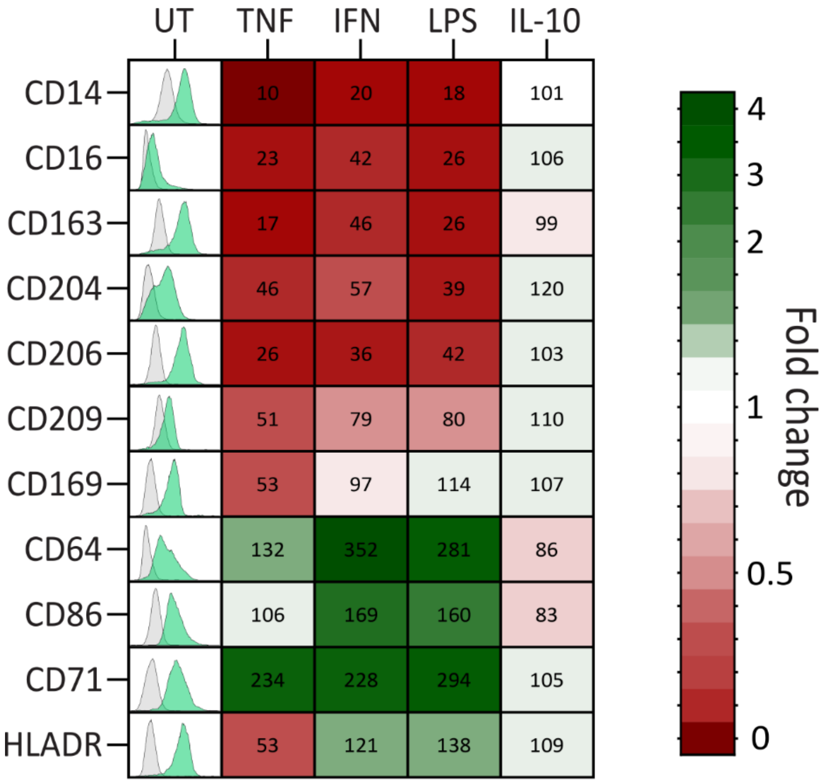

Figure S5: TNF, IFN- γand LPS depolarize NLC toward a M1-like phenotype. PBMC from CLL's patients were cultured in the presence of TNF or IFN- γor LPS or IL-10 during 14 days. The phenotype of NLC was analysis by flow cytometry. MFI ratio (marker/isotypic control) were normalized as percentage of change of marker signal in relation to basal signal – untreated conditions. Normalization has been performed for each sample separately, followed by calculation of mean of all experiments (8 donors).
(green: expression increased compared to UT; white: baseline expression in UT conditions; red: expression decreased compared to UT).
